# Safety and Efficacy of Immune Checkpoint Inhibitors in Human Immunodeficiency Virus-Associated Cancer: A Systematic Scoping Review

**DOI:** 10.3390/diseases13080230

**Published:** 2025-07-22

**Authors:** Ahmed D. Alatawi, Amirah B. Alaqyl, Reema J. Alalawi, Rahaf S. Alqarni, Razan A. Sufyani, Ghadi S. Alqarni, Raghad S. Alqarni, Jumana H. Albalawi, Raghad A. Alsharif, Ghada I. Alatawi, Elaf N. Albalawi, Danah A. Alanazi, Sultanah A. Naitah, Reem Sayad, Helal F. Hetta

**Affiliations:** 1 Department of Clinical Pharmacy, College of Pharmacy, Jouf University, Sakaka 72341, Saudi Arabia; adalatawi@ju.edu.sa; 2 College of Applied Medical Sciences, University of Tabuk, Tabuk 47315, Saudi Arabia; dr.ameerah2001@gmail.com (A.B.A.); alalawii.reema@gmail.com (R.J.A.); rhfffsq@gmail.com (R.S.A.); 3 College of Applied Medical Sciences, Umm Al-Qura University, Makkah 21955, Saudi Arabia; razansufyani@outlook.com; 4 PharmD Program, College of Pharmacy, University of Tabuk, Tabuk 47914, Saudi Arabia; ighadisaleh@gmail.com (G.S.A.); raghadalqarni08@gmail.com (R.S.A.); 421000067@stu.ut.edu.sa (J.H.A.); 421000501@stu.ut.edu.sa (R.A.A.); 451003299@stu.ut.edu.sa (G.I.A.); 451002774@stu.ut.edu.sa (E.N.A.); 421000390@stu.ut.edu.sa (D.A.A.); 5 PharmD Program, College of Pharmacy, Umm Al-Qura University, Makkah 21955, Saudi Arabia; sultananaitah@gmail.com; 6 Department of Histology, Faculty of Medicine, Assiut University, Assiut 71515, Egypt; reem.17289806@med.aun.edu.eg; 7 Division of Microbiology, Immunology and Biotechnology, Department of Natural Products and Alternative Medicine, Faculty of Pharmacy, University of Tabuk, Tabuk 71491, Saudi Arabia

**Keywords:** immune checkpoint inhibitors, programmed death-1, melanoma, lymphoma, small-cell lung cancer, non-small-cell lung cancer, immunodeficiency disease, human immunodeficiency virus

## Abstract

**Background/Objective:** People living with human immunodeficiency virus (PHIV) are at increased risk for malignancies, yet their access to immunotherapy remains limited due to concerns about safety and efficacy. This systematic scoping review evaluates the use of immune checkpoint inhibitors (ICIs) in HIV-associated cancers, analyzing patient outcomes, safety profiles, and the impact on HIV status. **Methods:** A comprehensive literature search was conducted in databases including PubMed, Scopus, Web of Science (WoS), and Medline, up to January 2025. Studies included assessing the efficacy of ICIs in cancer patients with HIV. The primary outcomes were (a) the efficacy of immune ICIs on prognosis, progression-free survival (PFS), and overall survival (OS). Secondary outcomes were the immune-related adverse events (irAEs) and the survival rate of cancer patients receiving ICIs. **Results:** A total of 107 cases from 19 studies published between 2011 and 2024 were reviewed. Responses to programmed death 1 (PD-1) inhibitors varied, with 27.1% achieving partial response, 23.36% experiencing stable disease, and 6.54% achieving complete response, while 34.57% had disease progression. Adverse events, including hematologic and endocrine toxicities, were common but mostly manageable. HIV viral loads remained stable in most cases. **Conclusions:** PD-1 inhibitors demonstrated potential efficacy in HIV-associated malignancies with a safety profile comparable to the general population. However, disease progression remained a concern, highlighting the need for optimized patient selection. Further well-controlled trials are essential to establish treatment guidelines and ensure equitable access to immunotherapy for PHIV.

## 1. Introduction

Several cancer types can be effectively treated with immune checkpoint inhibitors (ICIs), which are antibodies against cytotoxic T-lymphocyte-associated protein 4 (CTLA-4) or programmed death 1 (PD-1) [1,2,3,4,5]. The immunotherapeutic approach’s clinical success has validated the immune system’s function in cancer control and the fact that one of the characteristics of cancer is the neoplastic cells’ capacity to evade the immune system [6,7].

The United States Food and Drug Administration has approved two PD-1 inhibitors (nivolumab and pembrolizumab) and one programmed cell death-ligand 1 (PD-L1) inhibitor (atezolizumab) for previously treated non-small-cell lung cancer (NSCLC). Pembrolizumab is approved as a first-line therapy for NSCLC with elevated PD-L1 expression, and in combination with platinum and pemetrexed for nonsquamous NSCLC, regardless of PD-L1 expression levels [2,8,9,10]. Additionally, durvalumab, a PD-L1 inhibitor, is approved for use in patients with unresectable stage III NSCLC following chemoradiation [11]. As a result, nearly all patients with advanced NSCLC will probably be prescribed a PD-1 or PD-L1 inhibitor at some stage during their disease.

**Figure 1:**
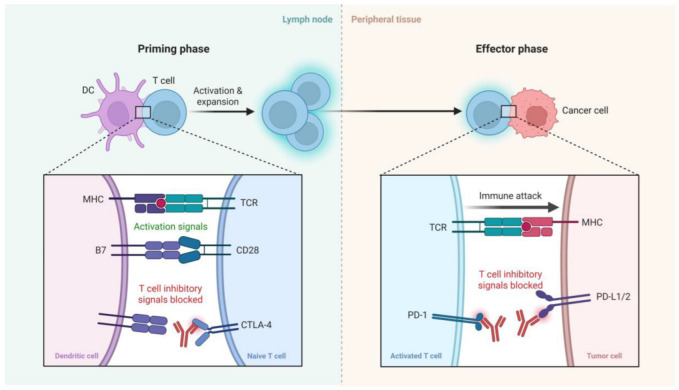
A schematic representation of the mechanism of action of immune checkpoint inhibitors. In the **priming phase** (**left**), dendritic cells (DCs) present antigens to naïve T cells, with costimulatory signals provided via CD28-B7 interactions. CTLA-4 competes with CD28, providing inhibitory signals that can be blocked by CTLA-4 inhibitors. In the **effector phase** (**right**), activated T cells recognize tumor antigens via MHC-TCR interactions. PD-1 on T cells binds to PD-L1/2 on tumor cells, leading to T cell exhaustion, which is blocked by PD-1/PD-L1 inhibitors, restoring immune attack on tumor cells. Created with BioRender.com.

ICIs enhance antitumor immunity by targeting key regulatory pathways that normally limit T cell activation and function. As illustrated in Figure 1, ICIs exert their effects during two critical phases of the immune response: the priming phase and the effector phase. In the priming phase, dendritic cells (DCs) present tumor antigens to naïve T cells in lymph nodes. This process requires both antigen-specific signals via the interaction of major histocompatibility complex and T cell receptor (MHC–TCR) and costimulatory signals through CD28–B7 engagement. However, CTLA-4, an inhibitory receptor on T cells, competes with CD28 for B7 binding, thereby dampening T cell activation. CTLA-4 inhibitors block this negative regulation, promoting full T cell activation and clonal expansion. In the effector phase, activated T cells recognize tumor antigens presented by MHC on cancer cells. Here, the PD-1 receptor on T cells interacts with its ligands PD-L1 or PD-L2 expressed on tumor cells, leading to T cell exhaustion and immune evasion. PD-1/PD-L1 inhibitors block this interaction, thereby restoring T cell function and enabling an effective immune attack on tumor cells. Collectively, ICIs reinvigorate antitumor T cell responses by blocking inhibitory checkpoints in distinct stages of the immune response [12,13,14,15] (Figure 1).

However, patients receiving immunotherapy need to be closely watched for the emergence of toxicities because these medications can be linked to severe and sometimes lethal immune-related adverse events (irAEs) [16,17,18,19]. Remarkably, in patients with advanced melanoma and NSCLC, the incidence of irAE has been associated with an increased antitumor efficacy of anti-PD-1 therapy [20,21,22].

Patients with a history of autoimmune disease (AID) have typically been excluded from cancer clinical studies of ICIs due to concerns that they may be more susceptible to severe irAEs [2,8]. The ICI is now frequently used to treat individuals with mild-to-moderate pre-existing AID. According to recent research, AID patients may benefit from anti-CTLA-4 and anti-PD-1 antibodies [23,24,25]. The adverse events of ICIs in patients with advanced melanoma and a history of AID have been assessed in two retrospective studies. Of the 30 melanoma and AID patients treated with ipilimumab, 27% had an aggravation of their pre-existing AID, 33% suffered grade 3 to 5 adverse events, and one patient died from colitis as a result of the medication [23]. In contrast, 52 patients with melanoma and AID who used PD-1 inhibitors appeared to have lesser toxicities; 38% of patients experienced an AID flare and 29% of patients experienced irAEs (10% grade 3 and no grade 4 or 5 irAEs) [24]. Both of these studies indicate that ICIs are generally safe to provide to patients with AIDS and melanoma, but the results might not apply to other cancers, because immunotherapy side effects can vary depending on the kind of tumor. For instance, a recent meta-analysis of immunotherapy research showed that NSCLC had a greater prevalence of pneumonitis linked to PD-1 inhibitors than melanoma [26]. All of the evidence that has been released thus far came from retrospective analyses of a limited number of patients. It is necessary to determine the advantages and disadvantages of ICIs in this population because individuals with AIDS are more likely to acquire lung cancer and other cancers.

We performed this systematic scoping review to investigate the safety and efficacy of immune checkpoint inhibitors in patients with cancer and a history of AIDS, as there is less information available regarding their usage.

## 2. Methods

### 2.1. Information Sources and Search Strategy

Considering the PRISMA (Preferred Reporting Items for Systematic Reviews and Meta-Analyses) extension for scoping reviews, a systematic scoping review of clinical trials was created [27]. This systematic review was registered on PROSPERO: CRD420251033070. Databases from SCOPUS, PubMed, the Web of Science (WoS), and MedLine through WoS were examined up to January 2025. The terminology ICIs refer to monoclonal antibodies that block the function of immunological checkpoints that serve as a brake on the immune system, such as receptor PD-1, which enhances the immune response to a tumor. Additionally, we included any type of cancer induced by human immunodeficiency virus (HIV), such as NSCLC, small-cell lung cancer (SCLC), melanoma, lymphoma, etc. These terms were used to review the case reports and case series published up to January 2025. Then, we used the Boolean operators AND and OR to search all databases. Details of the search strategy are mentioned in Supplementary File S1 (Table S1).

### 2.2. Eligibility Criteria

Inclusion criteria: Any type of cancer associated with HIV patients who received ICIs was included. Moreover, we included conference abstracts that published results from case reports and case series that met our inclusion criteria. Exclusion criteria: Experimental studies performed in vitro, animal studies, or reviews were not included.

### 2.3. Research Questions

This systematic scoping review aims to respond to the following question. In cancer patients associated with HIV, (a) what is the efficacy of ICIs on prognosis, progression-free survival (PFS), and overall survival (OS) as primary outcomes? (b) What are the irAEs of ICIs? (c) What is the survival rate of cancer patients receiving ICIs?

### 2.4. Trials Selection

After reading the abstracts and full texts, certain keywords prompted both researchers to choose the papers. The two researchers used the inclusion criteria to assess the studies. Subsequently, every abstract and full text was downloaded and evaluated independently based on the pre-established inclusion criteria. When there was disagreement among the authors, the third author assessed the acceptability of the study.

### 2.5. Data Extraction

Two authors independently reviewed and evaluated each full text that met the inclusion criteria to be included in this systematic scoping review. Each investigator independently created a table that included the most crucial details from the chosen trials, and the outcomes were compared. The table that summarizes the included studies lists the names of the authors, the publication year, the study design, the number of included cases, age, sex, diagnosis (cancer type, histology/stage), genotype, ART at enrolment, baseline CD4 T cell count (cells/μL), and plasma HIV viral load (copies/mL) at baseline.

According to the sheet of patient outcomes, it included previous lines of therapy, PD-1 inhibitors (name of drug, dose, route of administration, number of doses or cycles), other treatments combined with PD-1 inhibitors, duration of therapy (months), best of response (BOR) or progress, PFS (months), OS, immune-related, irAEs (toxicity) (grade), and alive or dead. Clinical and molecular biomarkers, such as CD4 T cell count (cells/μL) nadir, plasma HIV viral load (copies/mL) after treatment, and PD-L1 expression (when available), were systematically extracted from the included studies.

### 2.6. Outcome Measures

The primary outcomes were (a) the efficacy of immune checkpoint inhibitors on prognosis, PFS, and OS. Secondary outcomes were the irAEs of ICIs and the survival rate of cancer patients receiving immune checkpoint inhibitors.

The prognosis is presented in the forms of complete remission (CR), partial remission (PR), stable disease (SD), progressive disease (PD), PFS, and OS.

## 3. Results

### 3.1. Study Selection

**Figure 2:**
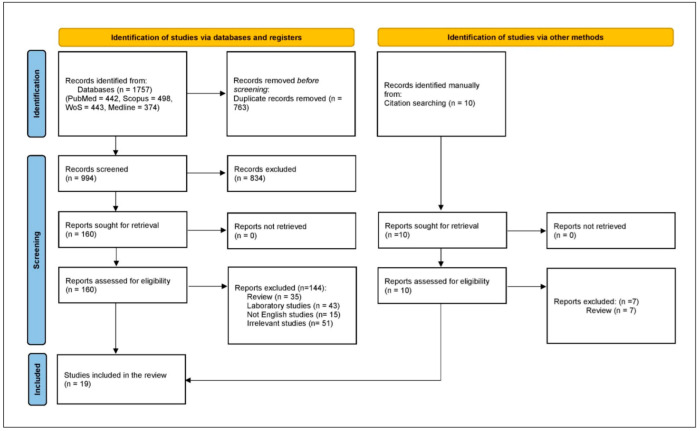
PRISMA flowchart.

There was an initial retrieval of 1757 studies, with 763 excluded as duplicates, leaving 994 for title and abstract screening. From these, 834 were excluded, and 144 were further excluded after full-text review, resulting in 16 case reports and case series, with 3 additional case series retrieved by manual search. Therefore, the systematic scoping review included 19 case reports and series (Figure 2).

### 3.2. Study Characteristics

This systematic scoping review included 107 cases from 19 studies, ranging from 2011 to 2024. This systematic scoping review also included 11 retrospective case series and 8 case reports [28,29,30,31,32,33,34,35,36,37,38,39,40,41,42,43,44,45,46]. The median age is 53 across studies, ranging from 29 to 85 years, with most patients being male (90.65%, n = 97/107), reflecting a known demographic bias in both HIV and cancer reporting. Only ten women were reported. The most common malignancies were lung cancer, either SCLC or NSCLC (36.44%, n = 39/107), lymphomas (21.49%, n = 23/107), and melanoma (13.08%, n = 14/107).

Antiretroviral therapy (ART) status was reported by 90.65% (n = 97/107), with 103 patients (95.4%) receiving ART at the time of ICI initiation. ART regimens varied across studies, with integrase strand transfer inhibitor (INSTI)-based combinations being the most common, followed by protease inhibitor (PI)-based and non-nucleoside reverse transcriptase inhibitor (NNRTI)-based regimens.

Baseline CD4+ T-cell counts were reported for 95 patients and ranged from 55 to 1147 cells/μL, with a median across studies typically between 200 and 450 cells/μL. Notably, several studies included patients with CD4 < 200 cells/μL, but outcomes were not always stratified by immunologic status. Plasma HIV viral load at baseline was reported in 84 cases. Most patients (n = 57, 53.2%) had undetectable viral loads, while a subset had low-level viremia (<400 copies/mL), and a few had high-level viremia (>100,000 copies/mL), underscoring the heterogeneity in virologic control at the time of immunotherapy initiation. Table 1 presents more details about the baseline characteristics of the patients included.

### 3.3. Patients’ Outcomes

This analysis included 107 cases from 19 studies, detailing the use of PD-1 inhibitors in patients. The most commonly administered PD-1 inhibitors were nivolumab (45.79%), pembrolizumab (32.7%), sintilimab (11.2%), camrelizumab (2.8%), and avelumab (1.86%), with treatment regimens varying in terms of dosage, duration, and combination therapies.

Most patients received PD-1 inhibitors as monotherapy, while some were treated in combination with chemotherapy, targeted therapy, radiotherapy, or immune-modulating agents. The duration of therapy varied significantly, ranging from a single cycle to over 40 cycles. Some patients discontinued therapy early due to disease progression or adverse events. More details of PD-1inhibitor treatment are presented in Table S2.

### 3.4. Efficacy Outcomes

**Figure 3:**
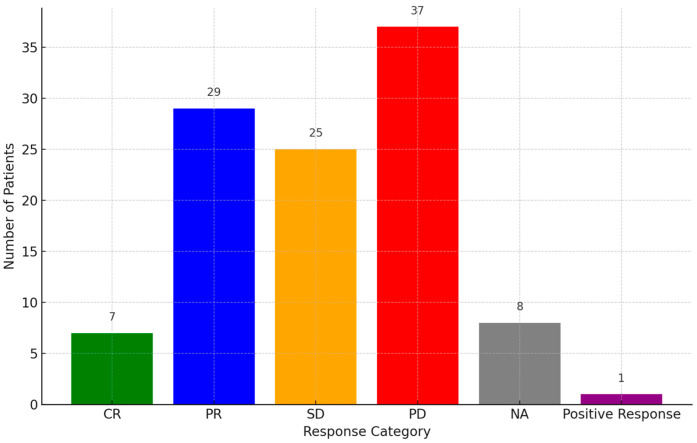
Response rate of patients to PD-1 inhibitors. **Abbreviations:** PR: partial response, PD: progressive disease, CR: complete response, SD: stable disease, NA: not assessed.

Responses to PD-1 inhibitors were categorized as PD (34.57%, 37/107), partial response (PR) (27.1%, 29/107), SD (23.36%, 25/107), or complete response (CR) (6.54%, 7/107), while eight cases were not assessed and one case gave a positive response (Figure 3). PR was observed in multiple cases, with some patients achieving durable responses beyond 24 months. A subset of patients demonstrated SD, indicating disease control, while others experienced disease progression despite treatment. The OS and PFS data were inconsistently reported across studies. More details of the prognosis of the disease are presented in Table S2.

**Table 1:** Baseline characteristics of the included cases. Abbreviations: NA: not assessed, ND: not detected, HAART: Highly Active Antiretroviral Therapy, HCC: hepatocellular carcinoma, SCC: small-cell carcinoma, NSCLC: non-small-cell lung cancer, ART: antiretroviral therapy. * Some studies considered the plasma HIV viral load (copies/mL) at baseline; when it is <20, it is an undetectable level. ** Data are presented in the form of the median and range. *** We included only one case that met our inclusion criteria.

Study ID	Study Design	Number of Included Cases	Age **	Sex (Number of Cases/Total)	Diagnosis (Cancer Type, Histology/Stage) (Number of Cases/Total)	Genotype (Number of Cases/Total)	ART at Enrolment (Number of Cases/Total)	Baseline CD4 T Cell Count (Cells/μL) **	Plasma HIV Viral Load (Copies/mL) at Baseline * (Number of Cases/Total)
Yazji et al., 2024 [28]	Retrospective case series	5	66 (41–70)	M (4/5) F (1/5)	NSCLC (III) (2/5) SCLC (IB) (1/5) Anal squamous cell cancer (IIA) (1/5) HCC (locally advanced) (1/5)	ND (5/5)	On ART (5/5)	301 (69–469)	Undetectable (4/5) NA (1/5)
Wu et al., 2023 [29]	Retrospective case series	15	44 (29–69)	M (15/15)	Large cell lung cancer (IV) (1/15) SCLC (IV) (4/15) Diffuse large B-cell lymphoma (II) (2/15) Diffuse large B-cell lymphoma (IV) (2/15) Burkitt’s lymphoma (IV) (2/15) Non-Hodgkin lymphoma (IV) (1/15) Hodgkin lymphoma (II) (1/15) Hodgkin lymphoma (IV) (1/15) Nasopharyngeal carcinoma (II) (1/15)	ND (15/15)	TDF + LAM + EFV (6/15) TDF + LAM + DTG (3/15) FTC + TAF + RAL (1/15) Not mentioned (3/15) TDF + DTG + FTC (1/15) BIC + FTC + TAF (1/15)	156 (55–375)	<40 (2/15) 198 (1/15) 2.18 × 10^5^ (1/19) Undetectable (1/15) NA (9/15)
Xiong et al., 2023 [30]	Retrospective case series	16 ***	29	M	Diffuse large B-cell lymphoma (Stage IIEa)	ND	On combination antiretroviral therapy	182	NA
Azizi et al., 2022 [31]	Case report	1	70	M	High-grade urothelial carcinoma of the left ureter metastatic to lymph nodes and lungs stage IV (T4 N2 M1)	ND	Darunavir, ritonavir, and raltegravir initially; later changed to dolutegravir, doravirine, and valacyclovir	NA	Undetectable
Idossa et al., 2022 [32]	Case report	2	(52–63)	M (2/2)	Metastatic castrate-resistant prostate cancer (2/2)	Mutation in AR T878A (allele frequency 1.3%) (1/2) BRCA2 mutation (T3310fs * 17, allele frequency 2.2%), TP53 R248W, TMPRSS2-ERG fusion (1/2)	on HAART (2/2)	261	Undetectable (2/2)
Alloghbi et al., 2021 [33]	Case report	1	60	M	Advanced cutaneous squamous cell carcinoma with metastasis.	ND	On HAART	NA	NA
Bertin et al., 2021 [34]	Reterospective case series	5	57.4	M (5/5)	NSCLC (adenocarcinoma, stage IV) (1/5) NSCLC (undifferentiated carcinoma, IV) (2/5) NSCLC (squamous carcinoma, IV) (2/5)	No EGFR/ALK (5/5)	On ART (4/5) Not yet (started later) (1/5)	NA	<30 (3/5) <31 (1/5) 44,500 (1/5)
Cesmeci et al.2021 [35]	Case report	1	50	M	Metastatic advanced-stage Kaposi Sarcoma	ND	On ART	450	Undetectable
Lau et al., 2021 [36]	Retrospective case series	3	56 (68–76)	M (3/3)	Merkel cell carcinoma (1/3) Metastatic melanoma (2/3)	ND (3/3)	TDF/3TC/EFV (2/3) EVG/COBI/TAF/FTC (1/3)	265 (323–468)	<20 (3/3)
Lurain et al., 2020 [47]	Retrospective case series	10	46 (34–67)	M (7/10) F (3/10)	Peritoneal primary effusion lymphoma (2/10) Germinal center B-cell-like diffuse large B-cell lymphoma (MYC-rearranged) (1/10) Extra cavity peritoneal primary effusion lymphoma (2/10) Aggressive B cell lymphoma (1/10) Non-germinal center B-cell-like diffuse large B-cell lymphoma (2/10) Pleural/pericardial/peritoneal primary effusion lymphoma (1/10) Plasmablastic lymphoma (1/10)	ND (10/10)	Not mentioned (10/10)	226 (107–557)	NA (10/10)
Bari et al., 2019 [38]	Retrospective case series	17	54 (40–62)	M (14/17) F (3/17)	Lung squamous cell carcinoma (II) (1/17) Lung squamous cell carcinoma (IV) (2/17) Lung adenocarcinoma (IV) (6/17) Mixed lung cancer (IV) (1/17) Anal squamous cell carcinoma (IV) (2/17) HCC (III) (1/17) HCC (IV) (1/17) Renal cell carcinoma (III) (1/17) Diffuse large B-cell lymphoma (IV) (1/17) Advanced basal cell carcinoma (1/17)	ND (17/17)	FTC/TDF + DTG (6/17) EVG/c/FTC/TDF (1/17) DTG, DRV/r (1/17) ABC/DTG/3TC (2/17) FTC/TDF + DRV (1/17) ETR, DTG, DRV/r (1/17) EFV/FTC/TDF (1/17) Not mentioned (1/17) RPV/FTC/TDF (1/17) TDF/RAL (1/17) DRV/c + DTG (1/17)	425.5 (150–795) NA (1/17)	<20 (5/17) <400 (3/17) 89 (1/17) 500 (1/17) NA (7/17)
Blanch-Lombarte et al., 2019 [39]	Case report	1	46	M	Metastatic melanoma amelanotic with axillary/pleural/vertebral metastases (IV)	BRAF V600 wild-type, NRAS/KIT wild-type	raltegravir + tenofovir/emtricitabine	<40	NA
Al Homsi et al., 2018 [40]	Case report	1	39	M	Merkel cell carcinoma, neuroendocrine tumor (IV)	ND	(standard HIV antiviral medications)	NA	110.672
Chang et al., 2018 [41]	Retrospective case series	16	65 (47–85)	M (16/16)	Anal squamous cell carcinoma (1/16) Combined HCC and pancreatic cancer (1/16) HCC (1/16) Hodgkin lymphoma (2/16) NSCLC (specific subtype not detailed) (2/16) NSCLC adenocarcinoma (4/16) NSCLC squamous cell carcinoma (2/16) Renal cell carcinoma (1/16) SCLC (1/16)	ND (16/16)	INSTI-based therapy (9/16) (PI-based therapy) (5/16) NNRTI-based therapy (1/16) No (elite controller) (1/16)	(164–304) NA (14/16)	NA (2/16) Undetectable (9/16) 1 log copies/Ml (2/16) 1.5 log copies/mL (2/16) 2 log copies/mL (1/16)
Galanina et al., 2018 [42]	Retrospective case series	9	44 (33–63)	M (9/9)	Kaposi’s sarcoma (cutaneous and LN) (T0I0S1) (4/9) Kaposi’s sarcoma (cutaneous w/lymphedema) (T1I0S1) (1/9) Kaposi’s sarcoma (GI involvement) (T1IS1) (1/9) Kaposi’s sarcoma (cutaneous, LN, lung) (T1I1S1) (1/9) Kaposi’s sarcoma (cutaneous, LN, bowel) (T1I1S1) (2/9)	KRAS Q61H, TP53 R273C (tissue), TP53 (ctDNA) (1/9) NF1 (ctDNA) (1/9) No alterations (1/9) None detected (4/9) PTPN6 M1 (1/9) TLL2 G465E (1/9)	On ART (9/9)	NA (9/9)	<20(1/9) <21 (1/9) 22 (1/9) 24 (1/9) 116,706 (1/9) 549,704 (1/9) ND (3/9)
Ostios-Garcia et al., 2018 [43]	Retrospective case series	7	52 (43–59)	M (6/7) F (1/7)	NSCLC, adenocarcinoma (7/7)	KRAS G12C (2/7) ND (4/7) KRAS G12V (1/7)	Started ART 3 weeks after PD-1 initiation (1/7), On ART before PD-1 therapy (6/7)	360 (57–1147)	12,589 copies/mL Undetectable (6/7)
Heppt et al., 2017 [44]	Retrospective case series	10	54.5 (30–74)	M (8/10) F (2/10)	Metastatic melanoma (9/10), Merkel cell carcinoma (1/10)	NRVX50/K (1/10) BRVK100E (4/10) Wildtype (4/10) CR (1/10)	On ART therapy (9/10) Not on ART therapy (1/10)	450 (76–870) Unknown (4/10)	20 (1/10) 3960 (1/10) Unknown (2/10) Undetectable (6/10)
Davar et al., 2015 [45]	Case report	1	47	M	Advanced melanoma, metastatic	IL28B polymorphism CT genotype; HCV genotype 1C	ART regimen included 2 nucleoside reverse transcriptase inhibitors and 1 non-nucleoside reverse transcriptase inhibitor	NA	NA
Burke et al., 2011 [46]	Case report	1	50	M	Metastatic melanoma	ND	On ART	320	Undetectable

### 3.5. Duration of Therapy and Treatment Patterns

Treatment duration ranged from a single cycle to over 40 cycles (median: 6 cycles). Pembrolizumab and nivolumab were the most commonly used ICIs. Combination regimens (e.g., with ipilimumab or pomalidomide) were also reported. Most patients were on suppressive ART with undetectable HIV viral loads during ICI therapy.

### 3.6. Survival Outcomes (PFS and OS)

Survival outcomes were heterogeneous. While several patients remained alive at the time of analysis (41.12%), others succumbed to disease progression or treatment-related complications. Long-term survival was observed in select cases, particularly among those with durable PR or SD.

PFS was explicitly reported in only 25 patients. Median PFS values ranged from 1 to 33 months, though not all cases reported time-to-event endpoints uniformly. Similarly, OS data were available for only a subset of patients, with many outcomes reported only as “alive” or “deceased” at last follow-up without exact durations.

A qualitative synthesis suggests prolonged survival (≥12 months) in many patients achieving disease control. However, due to the heterogeneity in cancer types, treatment regimens, and follow-up duration, a pooled survival estimate could not be generated. More details are presented in Table S2.

### 3.7. Immune-Related Adverse Events (irAEs)

**Figure 4:**
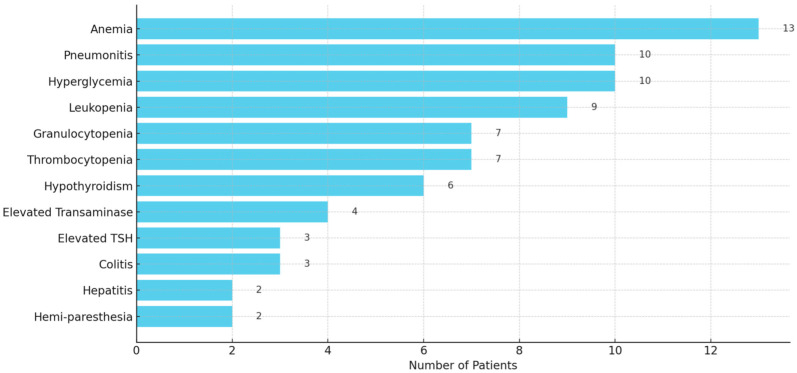
A horizontal bar graph displaying the frequency of reported immune-related adverse events (irAEs) among 107 patients. It clearly highlights the most common irAEs.

The irAEs were commonly reported in 65 out of 107 patients (60.7%), ranging in severity from mild (Grade 1) to life-threatening (Grade 4). The most frequently observed irAEs included anemia (13), pneumonitis (10), leukopenia (9), granulocytopenia (7), thrombocytopenia (7), elevated transaminase (4), colitis (3), hepatitis (2), hemi-paresthesia (2), and endocrinopathies such as hyperglycemia (10), hypothyroidism (6), and elevated thyroid stimulating hormone (TSH) (3) (Figure 4). Severe toxicity led to treatment discontinuation in several cases. More details of irAEs are presented in Table S2.

### 3.8. HIV-Related Parameters

CD4 counts were reported in 72 patients, with nadir values ranging from 15 to 1250 cells/μL. Viral load was undetectable in 67% of those with reported data. No clear association between baseline CD4 count or viral load and treatment efficacy or toxicity was observed. While many patients (67%) maintained undetectable viral loads, transient increases were noted in a few cases. CD4 counts varied widely, with some patients experiencing reductions during therapy. No significant HIV-related complications were reported, suggesting that PD-1 inhibitors were generally safe in this population. More details of CD4 T-cell count are presented in Table S2.

## 4. Discussion

We drew numerous significant conclusions from our study, which examined a sizable urban cohort of cancer cases linked to HIV that could influence clinical management and healthcare policy. This systematic scoping review analyzed 107 cases from 19 studies published between 2011 and 2024. The most common malignancies included lung cancer (36.44%), lymphomas (21.49%), and melanoma (13.08%). Most patients (90.65%) were receiving ART at baseline. Most patients received nivolumab (45.79%) and pembrolizumab (32.7%), followed by sintilimab, camrelizumab, and avelumab.

While ICIs have demonstrated promising antitumor efficacy in the general population, their use in PHIV raises unique immunological considerations. HIV infection leads to chronic immune activation, CD4+ T-cell depletion, and functional impairment of cytotoxic T-lymphocytes, all of which can compromise effective antitumor immunity. Although ART restores CD4+ counts to some extent, immune exhaustion, marked by the sustained expression of inhibitory receptors such as PD-1 on T cells, remains prevalent in PHIV [48]. This suggests a theoretical rationale for ICI benefit, particularly PD-1/PD-L1 blockade, in reactivating HIV-exposed or tumor-specific T cells [49]. However, the degree to which HIV-related immune dysfunction impacts ICI efficacy or toxicity is not fully understood. Emerging data from small cohorts and retrospective analyses indicate that ICIs are generally well tolerated in PHIV with controlled viremia and adequate CD4+ counts, but larger, prospective studies are needed to confirm these findings and optimize patient selection and monitoring [50].

HIV-associated malignancies may differ immunologically and biologically from their counterparts in immunocompetent individuals, potentially affecting the response to ICIs. The underlying chronic immune activation, persistent T-cell dysfunction, and incomplete immune restoration, even with ART, can alter antitumor immune responses. Moreover, many HIV-associated cancers are linked to oncogenic viruses (e.g., Kaposi sarcoma with HHV-8, certain lymphomas with EBV), which may influence tumor immunogenicity and the expression of checkpoint molecules such as PD-L1. These viral antigens could either serve as immune targets or contribute to immune evasion. Early evidence suggests that while some cancers in PHIV respond similarly to ICIs as in HIV-negative individuals, differences in tumor microenvironment composition, such as lower CD8+ T-cell infiltration or differential PD-L1 expression, might contribute to varied therapeutic outcomes. Therefore, understanding the unique immunopathology of HIV-associated cancers is crucial to optimizing immunotherapy in this population [51,52].

Individuals with HIV-related lung cancer were often detected in an advanced stage. The poor screening rates in this demographic and the early age of diagnosis, which preclude many from screening, result in PHIV being detected in an advanced stage. The United States Preventive Services Task Force (USPSTF) lung cancer screening criteria in 2021 include a lower age cutoff (50 years) and pack years (20 pack-years), which greatly increases the eligible population [53]. However, these criteria are still insufficient for the early detection of lung cancer in PHIV. There are systemic barriers to lung cancer screening in PHIV. These include limited access to healthcare, insurance disparities, HIV-related stigma within healthcare settings, and lower socioeconomic status—all of which may reduce engagement in preventive services. Furthermore, the current USPSTF lung cancer screening guidelines focus primarily on age and smoking history and may not adequately account for HIV-related immunosuppression and chronic inflammation as independent risk factors. This may further disadvantage PHIV by underrepresenting them in screening eligibility criteria. These intersecting barriers highlight the need for tailored screening strategies and inclusive policy revisions.

Based on USPSTF criteria from 2013 to 2016, the proportion of eligible PHIV individuals, as reported by Cali Daylan et al. (2024), who acquired a lung cancer diagnosis through routine screening, was quantitatively lower than that of the HIV-negative cohort [54]. Due to the age restriction, 20% (8/40) of the PHIV cohort were disqualified from lung cancer screening [54]. PHIV may benefit from a lower age cutoff, so bigger cohorts should be used to study the best lung cancer screening criteria.

Historically, PHIV was routinely excluded from clinical trials involving ICIs due to concerns about irAEs, viral reactivation, and confounding effects of immunosuppression. These exclusions were often present in early trial protocols and not always supported by empirical data. Regulatory agencies have begun to address this issue. For example, in 2020, the FDA issued draft guidance recommending that PHIV should not be categorically excluded from cancer clinical trials and encouraged inclusion where scientifically and ethically appropriate [55]. Similarly, the European Medicines Agency (EMA) has emphasized the importance of broadening eligibility criteria in oncology trials to improve generalizability and equity. Additionally, our review provides empirical evidence suggesting that ICIs can be both effective and tolerable in PHIV with cancer, especially when patients are on stable ARTs and have controlled viral loads. These findings support the movement toward more inclusive trial designs and may inform future revisions of eligibility criteria to reduce the unnecessary exclusion of PLHIV from immunotherapy trials.

Although data are still emerging, most studies to date indicate that HIV-positive individuals with cancer exhibit comparable clinical outcomes to HIV-negative patients when treated with ICIs [52]. On the other hand, studies have shown that PHIV with lung cancer tends to experience higher mortality rates and poorer survival outcomes compared to HIV-negative counterparts, even after adjusting for cancer stage and treatment [56,57]. Small prospective studies and retrospective case series have shown similar response rates in NSCLC, melanoma, and Kaposi sarcoma, suggesting that well-controlled HIV infection does not preclude a benefit from ICIs. However, certain factors such as low CD4+ counts, high HIV viral load, and altered immune profiles may affect treatment efficacy in some patients. Additionally, the exclusion of PLWH from many pivotal trials has resulted in a lack of robust comparative data, highlighting the need for inclusive trial designs and stratified analyses [2,8].

While PD-1 inhibitors demonstrated efficacy in a subset of patients, over one-third experienced disease progression. However, PD-1 inhibitors showed promising efficacy in certain patients, particularly those achieving PR or SD. PD-1 inhibitors (e.g., nivolumab, pembrolizumab) bind to the PD-1 receptor on T cells, blocking interaction with its ligands (PD-L1 and PD-L2), which may restore exhausted HIV-specific T cell function and impact latent viral reservoirs [58,59]. PD-L1 inhibitors (e.g., atezolizumab, durvalumab) block the PD-L1 ligand, potentially resulting in a more limited effect on systemic T cell reinvigoration.

Recent studies highlight the heterogeneity of PD-L1 expression across tumor types and patient populations, emphasizing that PD-L1 status alone may not be a reliable predictor of response to ICIs in all contexts. For instance, melanoma research has demonstrated that PD-L1 expression exhibits both spatial and temporal variability, and its predictive value is influenced by tumor-intrinsic and immune microenvironment factors [60,61]. Furthermore, intracellular signaling pathways, such as those involving NF-κB and PI3K/Akt/mTOR, have been implicated in PD-L1 upregulation, independent of interferon signaling [62,63], further complicating its role as a biomarker.

The dysregulation of T cells raises the risk of cancer and may affect treatment as well. The depletion of CD4+ T cells in uncontrolled HIV promotes the growth of virus-infected cells, which in turn encourages the development of malignancies linked to the virus. Chronic antigen exposure in HIV infection causes T cells to become fatigued and express more immunological checkpoints, such as PD-1, even when CD4 numbers are within the normal range. T-cell function and proliferation are hindered by this alteration [47]. Furthermore, the majority of research indicates that uncontrolled HIV infection results in a decreased TCR repertoire, indicating that TCR diversity is disrupted in HIV infection [64]. It has been shown that TCR repertoire restoration requires more than just effective viral suppression with ART [65]. Cali Daylan et al. (2024) found that a tumor-infiltrating lymphocyte (TIL) distribution with HIV control and peripheral lymphocyte count offers fascinating new information that could be related to PHIV’s altered immune regulation [54].

Studying ICI responses in HIV-associated cancers can yield broader insights into how chronic viral infections impact antitumor immunity. These insights may be highly relevant to other virally driven malignancies, such as human papillomavirus (HPV)-associated head and neck or cervical cancers, and hepatitis C virus (HCV)-associated hepatocellular carcinoma. PHIV thus represents a valuable population for understanding the intersection of chronic viral infection, immune exhaustion, and therapeutic immune modulation—findings that may inform ICI use in other immunologically complex settings.

Significantly, 31% of HIV-related lung malignancies in our study exhibited peripheral CD4 levels below 200 cells/μL. This highlights the importance of enhancing access to and adherence to ART. Given that survival rates for HIV-associated lung cancer are comparable to those of the HIV-negative lung cancer population when subjected to identical standard care, prevention and early diagnosis are imperative [66].

Most of the included studies reported that patients were receiving stable ART regimens during ICI therapy. However, data regarding the direct interaction between specific ARTs and cancer proliferation or ICI efficacy were limited and inconsistently reported. While some preclinical studies suggest that certain ARTs (e.g., protease inhibitors) may have antitumor effects through the modulation of signaling pathways (e.g., PI3K/AKT), the clinical relevance of these findings remains unclear [67,68,69]. Additionally, no consistent evidence was found in the included studies to suggest that ART negatively impacts ICI efficacy or cancer progression.

Despite encouraging response rates to ICIs in PLHIV with cancer reported in our review, a notable proportion of patients still experience DP, highlighting critical gaps in our understanding of resistance mechanisms. Currently, no validated biomarkers exist to reliably predict ICI efficacy in this population. Traditional biomarkers such as PD-L1 expression and tumor mutational burden (TMB) have limited application due to inconsistent reporting and unknown relevance in the context of HIV-induced immune dysfunction. Moreover, host-related factors such as CD4+ T-cell count, immune exhaustion markers (e.g., PD-1), and ART history may significantly impact treatment outcomes. The development of HIV-specific biomarker frameworks is therefore essential to improving patient selection and therapeutic precision in this underserved population.

According to Cali Daylan et al. (2024) [54], PHIV often had lower PD-L1 scores than the HIV-negative population. This is interesting because another study found that PHIV had lower PD-L1 expression [69], while multiple studies found higher PD-L1 scores [70,71,72].

The equivalent rates in these trials indicate that the variance in PD-L1 expression is not associated with HIV viral suppression. It is important to note that the PD-L1 antibody E1L3N, rather than the 22C3 antibody, was utilized in the studies that reported higher PD-L1 scores in PHIV [54,71,72]. It is essential to acknowledge that multiple studies have demonstrated substantial concordance between E1L3N and 22C3 in detecting PD-L1 in NSCLC populations, suggesting that this may not be the primary factor contributing to the reported discrepancies, but this distinction may partially elucidate the variance [73,74]. Disparities between studies may be caused by differences in race/ethnicity, histology, and diagnostic stage.

Adverse events, particularly hematologic and endocrine toxicities, were common. However, PD-1 inhibitors did not appear to significantly compromise HIV control. The majority of occurrences were reversible with systemic glucocorticoid administration and were subsequently handled safely, aligning with findings of irAEs in HIV-uninfected populations, indicating that such medications can be utilized in perinatal HIV-infected individuals without significant treatment-related immune toxicities. Between 5% and 30% of individuals in the general population develop grade ≥ 3 irAEs [75]. Moreover, grade ≥ 3 irAEs were reported at rates of 15.1% for atezolizumab, 14.1% for nivolumab, 19.8% for pembrolizumab, and 28.6% for ipilimumab, as indicated by a systematic review and meta-analysis of data from 36 phase II and III randomized controlled trials (n = 15,370) involving HIV-uninfected cancer patients [76]. Zarif et al. (2023) demonstrated a relatively lower incidence rate of grade ≥ 3 irAEs (7.7%) at 6 months in individuals with PHIV [70]. This discrepancy might be explained by the first study’s retrospective design, which led to selection bias and the exclusion of some events. Wang et al. (2018) reported that the incidence of fatal ICI-associated adverse events was 0.7%, which is comparable to the 0.3% to 1.3% observed in HIV-uninfected people [77]. According to another trial, nivolumab was associated with a reduced incidence of irAEs than other ICIs [78]. Furthermore, viral infections like cytomegalovirus (CMV) may contribute to the remodeling of the immunological milieu surrounding the tumor, changing the host immune response and hence promoting the occurrence of irAEs [79].

A longer time after HIV diagnosis and a lower CD4 cell count were associated with a higher risk of severe treatment-related toxicity. Conflicting research exists about the influence of CD4+ T cell count on irAEs. Elevated CD4+ T cell counts before initiating ICI medication may correlate with an increased risk of irAEs, whereas alternative studies suggest that reduced CD4+ T cell counts in patients receiving ICI therapy may also be associated with a heightened risk of irAEs [70,80]. Consistent with the first hypothesis, individuals with a CD4 T cell count < 200/μL at the start of ICI have a greater risk of irAEs.

According to the literature, which advises stopping systemic medication in cases of grade 3–4 toxicity and moderate- to high-dose corticosteroid treatment, immunotherapy was typically stopped after the commencement of immune-mediated toxicities in the study [81,82].

### 4.1. Strength Points

This study systematically analyzes 19 studies spanning over a decade (2011–2024), providing a broad and representative evaluation of PD-1 inhibitors in HIV-associated malignancies. The inclusion of various malignancies (lung cancer, lymphomas, melanoma) allows for a more comprehensive understanding of treatment responses across different tumor types in PHIV. The study highlights the need for tailored lung cancer screening criteria for PHIV, emphasizing the limitations of current USPSTF recommendations and their impact on early detection. Findings suggest that PD-1 inhibitors can be effective in a subset of patients, which informs treatment strategies. Moreover, this study confirms that PD-1 inhibitors do not significantly compromise HIV control, supporting their use in this population while highlighting manageable irAEs. By comparing irAE rates in HIV-infected and -uninfected populations, the study provides crucial data on the relative safety of ICIs in PHIV, reinforcing their feasibility in this group.

### 4.2. Limitations and Recommendations

The studies included vary in design, sample size, and reporting standards, leading to inconsistencies in outcome assessment (e.g., overall survival and progression-free survival). Due to inconsistent survival reporting, it remains unclear how PD-1 inhibitors impact long-term prognosis in PHIV. This heterogeneity in study designs, outcome measures, and reporting across the included studies is considered a limitation of our review. Moreover, the descriptive nature of the review, small sample sizes, and limited availability of controlled comparisons need an interpretation with caution.

All included studies are retrospective, increasing the likelihood of selection bias and limiting the ability to establish causality. This study predominantly includes male participants, potentially limiting the generalizability to female and transgender PHIV populations. All data is derived from retrospective series and case reports, which reduces the strength of the evidence compared to randomized controlled trials. Most included cases originated from high-income countries, with very few reports from low- and middle-income countries (LMICs). Additionally, several studies lacked sufficient geographic identifiers or regional context, preventing meaningful comparative analysis. Therefore, there is a need for more data from underrepresented regions to better understand global disparities in access to and outcomes of ICI therapy among people living with HIV.

Differences in PD-1 antibody assays and patient demographics (e.g., histology) may contribute to conflicting findings regarding PD-1 expression in PHIV. The relationship between CD4 count and irAEs remains inconclusive, warranting further investigation. Moreover, the majority of included cases involved PD-1 inhibitors, with only a few reporting the use of PD-L1 inhibitors. Therefore, there is a need for further studies comparing the clinical and immunological outcomes of PD-1 versus PD-L1 blockade in PHIV.

Therefore, more robust, well-controlled trials are essential to establish definitive guidelines for the use of immunotherapy in HIV-associated cancers and to ensure equitable access to effective cancer treatments for PHIV. Stratified randomization by baseline CD4 count, HIV viral load, and cancer type would significantly improve the statistical rigor of future studies assessing ICI safety and efficacy in PHIV. Such stratification could help delineate how immunologic status—particularly CD4 counts < 200 cells/μL—influences treatment outcomes and the risk of irAEs. Additionally, stratification would allow for more accurate subgroup analyses, enabling the identification of populations who may derive the most benefit or experience higher toxicity. Moreover, a minimum follow-up duration of 12–24 months would be important to capture long-term efficacy, the durability of response, and delayed immune-related adverse events.

Further research is warranted to elucidate how HIV-related immune exhaustion, characterized by the progressive depletion and functional impairment of lymphocytes, particularly CD4+ and CD8+ T cells, may influence the efficacy of ICIs. Multi-omics approaches hold great promise in disentangling HIV-specific immune alterations from tumor-specific immune responses to ICIs. For example, spatial transcriptomics could provide the high-resolution mapping of tumor-immune niches, revealing how chronic inflammation and immune cell localization differ in PHIV. TCR clonality assays can distinguish clonal expansions of HIV-specific versus tumor-specific T cells, offering insights into immune repertoire skewing. Additionally, single-cell RNA sequencing combined with proteomics could help characterize exhausted immune phenotypes and identify molecular signatures associated with co-infections such as CMV [83,84,85,86]. These approaches could advance biomarker discovery and optimize patient stratification in future ICI trials for PHIV.

Longitudinal studies examining the timing and mechanisms of immune dysregulation in PHIV could provide critical insights into optimal treatment windows and the identification of predictive biomarkers for response to immunotherapy. Longer follow-up periods (≥24 months) are necessary to capture delayed immune-related toxicities and disease recurrence, which may be underrepresented in current short-term data. Moreover, there is a need for future studies to investigate potential pharmacological interactions between ART and cancer immunotherapy. Given the heterogeneity in ICI regimens, cancer types, and ART management among PHIV, future research would benefit from prospective trial designs that can accommodate this complexity. Adaptive and platform trial frameworks offer distinct advantages over retrospective analyses by allowing for real-time modifications based on interim outcomes, stratification by key clinical variables (e.g., ART adherence, baseline CD4+ count, HIV viral load), and the simultaneous evaluation of multiple interventions. These designs can mitigate retrospective bias and generate more generalizable and nuanced evidence, ultimately facilitating the development of tailored immunotherapeutic strategies for PHIV.

One of the major challenges we observed was the inconsistency in how HIV-related variables were reported across studies. Important clinical parameters—such as nadir and current CD4+ T-cell counts, HIV viral load, duration and type of ART, and history of AIDS-defining illnesses—were often underreported or inconsistently documented. This limits the ability to conduct meaningful subgroup analyses and to evaluate potential modifiers of ICI safety and efficacy in PHIV. Similarly, the grading of irAEs varied across studies, with some not explicitly using standardized criteria such as the Common Terminology Criteria for Adverse Events (CTCAE). These issues hinder cross-study comparability and weaken the strength of pooled analyses.

To address these gaps, future prospective studies and clinical trials should prioritize the inclusion of standardized HIV-specific metrics and consistent irAE grading. This would not only enhance the internal validity of individual studies but also facilitate data pooling across studies. Initiatives such as the CATCH-IT consortium (Cancer Therapy Using Checkpoint Inhibitors in HIV) exemplify the importance of collaborative, harmonized data collection in this field. Furthermore, adapting existing CONSORT guidelines for immunotherapy trials—such as the CONSORT-immune extension—to better reflect the needs of PHIV could significantly improve the quality and transparency of future research [87].

### 4.3. Clinical Implications

Despite encouraging preliminary findings, the generalizability of current evidence remains limited by the underrepresentation of key demographic and geographic populations. Most studies to date have been conducted in high-income countries and include predominantly male participants, leaving critical gaps in our understanding of ICI safety and efficacy in women, transgender individuals, and people in LMICs—regions that bear a disproportionate burden of HIV-associated cancers. Addressing these gaps will require deliberate and equity-oriented strategies. Decentralized clinical trial models, which allow for remote enrollment, monitoring, and follow-up, could reduce geographic and logistical barriers to participation. Strategic partnerships with HIV and oncology clinics in LMICs, combined with investments in local research infrastructure and capacity-building, are essential for enabling inclusive and sustainable clinical research. Additionally, future studies should incorporate prespecified targets for the enrollment of underrepresented groups and ensure culturally sensitive, community-engaged recruitment approaches. These efforts are crucial for ensuring that immunotherapy research reflects the diversity of the global HIV population and for informing treatment guidelines that are both inclusive and globally relevant.

In addition to the clinical and policy implications of our findings, this review also highlights key biological questions that remain unanswered and merit further mechanistic investigation. While retrospective clinical data provide important early signals of safety and efficacy, advancing the field to meet the threshold of biological insight required for transformative progress will depend on the deeper interrogation of underlying mechanisms. Specifically, mechanistic studies employing in vivo models of HIV-associated tumors could offer controlled systems to investigate how chronic viral infection, ART-induced immune reconstitution, and tumor immunogenicity interact to influence ICI responses. Moreover, high-dimensional techniques such as single-cell RNA sequencing, mass cytometry, and spatial transcriptomics could help identify specific immune cell subsets involved in both therapeutic response and irAEs. The profiling of TCR clonality, immune exhaustion markers, and cytokine networks in PHIV may also reveal novel biomarkers predictive of ICI efficacy or toxicity. These approaches would allow researchers to disentangle HIV-related immune dysregulation from cancer- and therapy-specific immune dynamics, thus providing causal insight that complements and extends our clinical synthesis. Our review highlights the urgent need for such mechanistic studies, which are critical for translating observational signals into actionable immunologic understanding.

## 5. Conclusions

This review highlights the potential efficacy and safety of PD-1 inhibitors in HIV-associated malignancies. While some patients showed durable responses, many experienced disease progression, emphasizing the need for better patient selection. The safety profile was comparable to the general population, with manageable immune-related adverse events. Future well-controlled trials are crucial to refining treatment guidelines and ensuring equitable access to immunotherapy for PHIV.

## Data Availability

Not applicable.

## Supplementary Materials

The following supporting information can be downloaded at https://www.mdpi.com/article/10.3390/diseases13080230/s1. Table S1: Details of the search strategy for searching different databases; Table S2: Outcomes of the included patients. Abbreviations: PR: partial response, PD: progressive disease, CR: complete response, SD: stable disease, NA: not assessed.
