# Supplementary material for: Safety and Efficacy of Immune Checkpoint Inhibitors in Human Immunodeficiency Virus-Associated Cancer: A Systematic Scoping Review
Source: Diseases. 2025 Jul 22;13(8):230. doi: 10.3390/diseases13080230 (PMC12385830; doi:10.3390/diseases13080230)
Supplement: Supplementary file 1 [file diseases-13-00230-s001.zip › diseases-3645713-supplementary.pdf]

diseases

Systematic Review

Safety and Efficacy of Immune Checkpoint Inhibitors in Human Immunodeficiency Virus-Associated Cancer: A Systematic Scoping Review

Ahmed D. Alatawi [1], Amirah B. Alaqyl [2], Reema J. Alalawi [2], Rahaf S. Alqarni [2], Razan A. Sufyani [3], Ghadi S. Alqarni [4], Raghad S. Alqarni [4], Jumana H. Albalawi [4], Raghad A. Alsharif [4], Ghada I. Alatawi [4], Elaf N. Albalawi [4], Danah A. Alanazi [4], Sultanah A. Naitah [5], Reem Sayad [6], Helal F. Hetta [7*]

1 Department of Clinical Pharmacy, College of Pharmacy, Jouf University, Sakaka 72341, Saudi Arabia. adalatawi@ju.edu.sa
2 College of Applied Medical Sciences, University of Tabuk, Tabuk 47315, Saudi Arabia; dr.ameerah2001@gmail.com, Alalawii.reema@gmail.com, rhfffsq@gmail.com
3 College of Applied Medical Sciences, Umm Al-Qura University, Makkah 21955,, Saudi Arabia; RazanSufyani@outlook.com
4 PharmD Program, College of Pharmacy, University of Tabuk, Tabuk 47914, Saudi Arabia; Ighadisaleh@gmail.com, Raghadalqarni08@gmail.com, 421000067@stu.ut.edu.sa, 421000501@stu.ut.edu.sa, 451003299@stu.ut.edu.sa, 451002774@stu.ut.edu.sa, 421000390@stu.ut.edu.sa
5 PharmD Program, College of Pharmacy, Umm Al-Qura University, Makkah 21955,, Saudi Arabia; Sultana-naitah@gmail.com
6 Department of Histology, Faculty of Medicine, Assiut University, Assiut 71515, Egypt; reem.17289806@med.aun.edu.eg
7 Division of Microbiology, Immunology and Biotechnology, Department of Natural Products and Alternative Medicine, Faculty of Pharmacy, University of Tabuk, Tabuk 71491, Saudi Arabia; hhussen@ut.edu.sa, https://orcid.org/0000-0001-8541-7304

* Correspondence: Helal F. Hetta (hhussen@ut.edu.sa).

Academic Editor: Firstname Lastname

Received: date
Revised: date
Accepted: date
Published: date

Citation: To be added by editorial staff during production.

Copyright: © 2025 by the authors. Submitted for possible open access publication under the terms and conditions of the Creative Commons Attribution (CC BY) license (https://creativecommons.org/licenses/by/4.0/).

Table S1: Details of the search strategy for searching different databases.

**PubMed**	(Prolgolimab OR Nivolumab OR Pembrolizumab OR Atezolizumab OR Durvalumab OR Avelumab OR Cemiplimab OR Tislelizumab OR Dostarlimab OR Retifanlimab OR Programmed cell death protein 1 OR PD-1 Protein OR Programmed Cell Death 1 Receptor OR "Immune checkpoint inhibitors" OR "PD-1 inhibitors" OR "PD-L1 inhibitors" OR "CTLA-4 inhibitors" OR "checkpoint blockade therapy" OR "immune checkpoint blockade" OR "checkpoint inhibitors" OR "ipilimumab") AND ("cancer" OR "neoplasm" OR "malignancy" OR "solid tumors" OR "carcinoma" OR "lymphoma" OR "oncology" OR "Kaposi sarcoma" OR "NSCLC" OR "melanoma" OR "non-small cell lung cancer" OR "non small cell lung cancer") AND ("HIV" OR "Human Immunodeficiency Virus" OR "HIV-positive" OR "AIDS" OR PLWH OR MHIV OR WHIV OR "HIV-infected" OR "HIV-associated malignancies" OR "AIDS-related cancer")	**422**
**Scopus**	(Prolgolimab OR Nivolumab OR Pembrolizumab OR Atezolizumab OR Durvalumab OR Avelumab OR Cemiplimab OR Tislelizumab OR Dostarlimab OR Retifanlimab OR Programmed cell death protein 1 OR PD-1 Protein OR Programmed Cell Death 1 Receptor OR "Immune checkpoint inhibitors" OR "PD-1 inhibitors" OR "PD-L1 inhibitors" OR "CTLA-4 inhibitors" OR "checkpoint blockade therapy" OR "immune checkpoint blockade" OR "checkpoint inhibitors" OR "ipilimumab") AND ("cancer" OR "neoplasm" OR "malignancy" OR "solid tumors" OR "carcinoma" OR "lymphoma" OR "oncology" OR "Kaposi sarcoma" OR "NSCLC" OR "melanoma" OR "non-small cell lung cancer" OR "non small cell lung cancer") AND ("HIV" OR "Human Immunodeficiency Virus" OR "HIV-positive" OR "AIDS" OR PLWH OR MHIV OR WHIV OR "HIV-infected" OR "HIV-associated malignancies" OR "AIDS-related cancer")	**498**
**WoS**	(Prolgolimab OR Nivolumab OR Pembrolizumab OR Atezolizumab OR Durvalumab OR Avelumab OR Cemiplimab OR Tislelizumab OR Dostarlimab OR Retifanlimab OR Programmed cell death protein 1 OR PD-1 Protein OR Programmed Cell Death 1 Receptor OR "Immune checkpoint inhibitors" OR "PD-1 inhibitors" OR "PD-L1 inhibitors" OR "CTLA-4 inhibitors" OR "checkpoint blockade therapy" OR "immune checkpoint blockade" OR "checkpoint inhibitors" OR "ipilimumab") AND ("cancer" OR "neoplasm" OR "malignancy" OR "solid tumors" OR "carcinoma" OR "lymphoma" OR "oncology" OR "Kaposi sarcoma" OR "NSCLC" OR "melanoma" OR "non-small cell lung cancer" OR "non small cell lung cancer") AND ("HIV" OR "Human Immunodeficiency Virus" OR "HIV-positive" OR "AIDS" OR PLWH OR mniv OR whip OR "HIV-infected" OR "HIV-associated malignancies" OR "AIDS-related cancer") (Topic)	**443**
**MedLine**	(Prolgolimab OR Nivolumab OR Pembrolizumab OR Atezolizumab OR Durvalumab OR Avelumab OR Cemiplimab OR Tislelizumab OR Dostarlimab OR Retifanlimab OR Programmed cell death protein 1 OR PD-1 Protein OR Programmed Cell Death 1 Receptor OR "Immune checkpoint inhibitors" OR "PD-1 inhibitors" OR "PD-L1 inhibitors" OR "CTLA-4 inhibitors" OR "checkpoint blockade therapy" OR "immune	**374**

	checkpoint blockade" OR "checkpoint inhibitors" OR "ipilimumab") AND ("cancer" OR "neoplasm" OR "malignancy" OR "solid tumors" OR "carcinoma" OR "lymphoma" OR "oncology" OR "Kaposi sarcoma" OR "NSCLC" OR "melanoma" OR "non-small cell lung cancer" OR "non small cell lung cancer") AND ("HIV" OR "Human Immunodeficiency Virus" OR "HIV-positive" OR "AIDS" OR PLWH OR mniv OR whip OR "HIV-infected" OR "HIV-associated malignancies" OR "AIDS-related cancer") (Topic)	

Table S2: Outcomes of the included patients. Abbreviations: PR: Partial response, PD: Progressive disease, CR: Complete response, SD: Stable disease, NA: Not assessed.

**Study ID**	**Case order**	**Previous Lines of Therapy**	**PD-1 inhibitors (name of drug, dose, route of administration, number of doses or cycles)**	**Other treatments combined with PD-1 inhibitors**	**Duration of Therapy (months)**	**Best of Response or Progress**	**progression-free survival (PFS) (mo)**	**Overall survival (OS)**	**Immune-related Adverse Events (toxicity) (Grade)**	**CD4 T cell count (cells/μL) nadir**	**Plasma HIV viral load (copies/mL) after treatment**	**Alive or dead**
**Yazji et al. 2024 [28]**	Case 1	None	Pembrolizumab (q3w, 10 cycles), Switched to ipilimumab + nivolumab (3 cycles) due to disease progression	None	Pembrolizumab for 6 months; ipilimumab + nivolumab for 3 months	PR	NA	NA	None	NA	50	Dead
	Case 2	None	Pembrolizumab (q6w, 8 cycles)	None	11	SD	NA	NA	None	NA	Undetectable	Alive
	Case 3	None	Durvalumab (1 cycle)	None	one cycle only	PR	NA	NA	None	40	Undetectable	Dead

	Case 4	None	Nivolumab (3 cycles, q4w)	None	3	PR	NA	NA	None	NA	Undetectable	Dead
	Case 5	None	Nivolumab (40 cycles)	None	39	SD	NA	NA	None	69	Undetectable	Alive
	Case 1	Chemotherapy + targeted cancer therapy	Sintilimab (10 cycles)	None	7.2	SD	NA	NA	Pneumonitis (IV) Leukopenia (II) Hemiparesthesia (II) Granulocytopenia (I) Anemia (I) Thrombocytopenia (I) Hyperglycemia (I) Elevated transaminase (I) Convulsions, dizziness	159	NA	Dead
**Wu et al. 2023 [29]**	Case 2	Chemotherapy + targeted cancer therapy	Sintilimab (6 cycles, ongoing)	None	5.1	PD	NA	NA	Anemia (II)	137	Undetectable	Alive

									Thrombocytopenia (II)<br>Leukopenia (I) Hyperglycemia (I)			
	Case 3	Chemotherapy + surgery	Sintilimab (34 cycles, ongoing)	None	27.8	SD	NA	NA	Leukopenia (IV)<br>Granulocytopenia (IV)<br>Thrombocytopenia (III)<br>Dyskinesia (III)<br>Septicemia (III)<br>Anemia (II)<br>Pneumonitis (II)<br>Rash (II)<br>Hyperglycemia (I)	177	NA	Alive
	Case 4	Chemotherapy	Sintilimab (2 cycles, refused further treatment)	None	18.3	SD	NA	NA	Anemia (I)<br>Mouth ulcers (I)	NA	NA	Alive

	Case 5	Chemotherapy	Sintilimab (3 cycles, refused further treatment)	None	Not mentioned	PD	NA	NA	Anemia (II) Nausea (II) Vomiting (II) Hypoglycemia (I) Hypokalemia (I) Hemiparesthesia (I)	NA	NA	Not reported
	Case 6	Chemotherapy + targeted therapy + iNKT cell therapy	Sintilimab (2 cycles)	None	1.9	SD	NA	NA	Septicemia (IV) Anemia (III) Hyperglycemia (I) Diarrhea (I)	NA	NA	Dead
	Case 7	Chemotherapy	Sintilimab (31 cycles, ongoing)	None	26.1	SD	NA	NA	Hyperglycemia (I)	337	NA	Alive
	Case 8	Chemotherapy + targeted therapy	Sintilimab (4 cycles, ongoing)	None	3.8	PD	NA	NA	Anemia (III), Septicemia (III) Leukopenia (II) Granulocytopenia (I) Thrombocytopenia (I)	NA	43.8	Alive

									Thrombocytopenia Hyperglycemia (I)			
	Case 9	Chemotherapy + targeted therapy + radiotherapy	Sintilimab (3 cycles)	None	3.7	SD	NA	NA	Septicemia (IV) Anemia (III) Nausea (II) Hyperglycemia (I) Fatigue (I) Hypokalemia (I)	NA	NA	Dead
	Case 10	Chemotherapy	Nivolumab (4 cycles, refused further treatment)	None	39.9	NA	NA	NA	Leukopenia (I) Granulocytopenia (I)	824	NA	Alive
	Case 11	Chemotherapy + targeted cancer therapy	Sintilimab (2 cycles, ongoing)	None	1.7	PD	NA	NA	Leukopenia (IV) Granulocytopenia (III) Anemia (III) Thrombocytopenia (II) Hyperglycemia (II)	NA	NA	Alive

									Soft tissue infection (II) Rash (II) Hematochezia (I)			
	Case 12	Chemotherapy + targeted therapy + radiotherapy	Sintilimab (1 cycle)	None	1.2	NA	NA	NA	Anemia (IV) Septicemia (IV) Urinary tract infection (I)	NA	NA	Dead
	Case 13	Chemotherapy + radiotherapy	Sintilimab (9 cycles, ongoing)	None	6.3	SD	NA	NA	Anemia (II) Rash (II) Hearing loss (II) Leukopenia (I) Elevated transaminase (I) Fatigue (I)	288	NA	Alive
	Case 14	Chemotherapy	Camrelizumab (1 cycle, ongoing)	None	1.7	NA	NA	NA	Weight loss (II) Fatigue (I) Pneumonitis (I)	NA	NA	Alive
	Case 15	Chemotherapy	Camrelizumab (7 cycles)	None	6.2	PR	NA	NA	Anemia (IV)	206	NA	Dead

									Septicemia (IV) Granulocytopenia (III) Dyskinesia (III) Leukopenia (II) Elevated transaminase (II) Weight loss (II)			
**Xiong et al. 2023 [30]**	Case 4	R-EPOCH × 4 cycles , RR × 4 cycles	Camrelizumab (16 cycles)	Lenalidomide × 6 months	Not mentioned	PD	NA	NA	None	NA	NA	Not reported
**Azizi et al. 2022 [31]**	Case 1	Chemotherapy (carboplatin and gemcitabine) followed by pembrolizumab.	Pembrolizumab, 7 cycles, followed by EV, initially at 1.25 mg/kg intravenously on days 1, 8, and 15 out of a 28-day cycle. The dose was reduced to 0.75 mg/kg due to toxicity and then increased to	None	Not mentioned	PR	NA	NA	Severe toxicity, including generalized pruritic rash, thrush, mucositis, anorexia, diarrhea, colitis, acute kidney injury, and pancytopenia	NA	Undetectable	Alive

			1 mg/kg after cycle 2									
**Idossa et al. 2022 [32]**	Case 1	Not mentioned	Pembrolizumab (36 cycles)	A triple (efavirenz-emtricitabine-tenofovir) with Raltegravir	31	PR	NA	Alive at the time of analysis	None	NA	Undetectable	Alive
	Case 2	Not mentioned	Pembrolizumab (19 cycles)	Descovy (emtricitabine/tenofovir alafenamide) with Dolutegravir	14	SD	NA	Deceased	None	NA	Undetectable	Dead
**Alloghbi et al. 2021 [33]**	Case 1	Mohs surgery, chemo-radiotherapy with cisplatin (later switched to carboplatin due to acute kidney injury).	Cemiplimab; 33 cycles.	None	24	CR	NA	NA	None	NA	NA	Alive
**Bertin et al. 2021 [34]**	Case 1	None	Pembrolizumab (200 mg, 5 cycles)	None	Not specified	PD	NA	9	None	413	<30	Dead
	Case 2	None	Pembrolizumab (200 mg, 7 cycles)	None	Not specified	PR	NA	8	Hyperthyroidism (I)	1000	<30	Dead
	Case 3	None	Pembrolizumab (200 mg, 1 cycle)	None	Not specified	PD	NA	3	None	463	<30	Dead
	Case 4	None	Pembrolizumab (200 mg, 28 cycles)	None	Not specified	PR	NA	25	Thrombocytopenic purpura (III)	76	<30	Alive

	Case 5	None	Pembrolizumab (200 mg, 4 cycles)	None	Not specified	PD	NA	4	None	1024	<30	Dead
**Cesmeci et al.2021 [35]**	Case 1	Not mentioned	Pembrolizumab, 200 mg IV q3w (6 cycles)	None	6	PR	NA	Still alive at follow-up	None	NA	Undetectable	Alive
	Case 1	Carboplatin and etoposide	Avelumab (anti-PDL1), 10 mg/kg, q2w	None	8	PR with PD	NA	Died	None	53	NA	Dead
	Case 2	Dabrafenib and trametinib	Nivolumab (anti-PD1) 3 mg/kg + Ipilimumab (anti-CTLA-4) 1 mg/kg, q3w	Dabrafenib and trametinib	4	PD	NA	Died	None	210	NA	Dead
**Lau et al. 2021 [36]**	Case 3	Radiotherapy	Nivolumab (anti-PD1) 3 mg/kg + Ipilimumab (anti-CTLA-4) 1 mg/kg, q3w	Radiotherapy	Ongoing maintenance therapy	PR with SD	NA	Ongoing	None	125	NA	Alive
	Case 1	Dolutegravir, Tenofovir Alafenamide Fumarate, Emtricitabine	Pembrolizumab (13 cycles)	None	Not mentioned	PR	NA	13	None	NA	NA	Dead
**Lurain et al. 2021 [47]**	Case 2	Dolutegravir, Tenofovir Disoproxil	Pembrolizumab (7 cycles)	None	Not mentioned	SD	11	11	Hypothyroidism (II)	NA	NA	Dead

		Fumarate, Emtricitabine										
	Case 3	Darunavir, Ritonavir, Dolutegravir, Tenofovir Disoproxil Fumarate, Emtricitabine	Pembrolizumab (6 cycles)	None	Not mentioned	SD	15	15	Hypothyroidism (II)	NA	NA	Alive
	Case 4	Darunavir, Cobicistat, Dolutegravir, Tenofovir Alafenamide Fumarate, Emtricitabine	Pembrolizumab (15 cycles)	None	Not mentioned	PR	33	33	None	NA	NA	Alive
	Case 5	Dolutegravir, Tenofovir Alafenamide Fumarate, Emtricitabine	Pembrolizumab (6 cycles)	None	Not mentioned	PR	NA	15	None	NA	NA	Dead
	Case 6	Dolutegravir, Abacavir, Lamivudine	Pembrolizumab (7 cycles) + pomalidomide (12 cycles)	None	Not mentioned	SD	24	24	Guillain-Barre syndrome (III)	NA	NA	Alive
	Case 7	Dolutegravir, Tenofovir Alafenamide Fumarate, Emtricitabine	Pembrolizumab (22 cycles) + pomalidomide(19 cycles)	None	Not mentioned	CR	NA	21,Ongoing	Hepatitis (III)	NA	NA	Alive
	Case 8	Bictegravir, Tenofovir Alafenamide Fumarate, Emtricitabine	Pembrolizumab (3s cycle)	None	Not mentioned	PD	3	3	None	NA	NA	Dead
	Case 9	Bictegravir, Tenofovir Alafenamide Fumarate, Emtricitabine	Pembrolizumab(4 cycles)+	None	Not mentioned	PD	4	4	None	NA	NA	Dead

			pomalidomide (1 cycle)									
	Case 10	Bictegravir, Tenofovir Alafenamide Fumarate, Emtricitabine	Pembrolizumab (7 cycles) + pomalidomide (7 cycles)	None	Not mentioned	PR	9	9	None	NA	NA	Alive
	Case 1	Not mentioned	Atezolizumab, 3 doses	Carboplatin and Paclitaxel	Not mentioned	PD	NA	NA	None	NA	NA	Dead
	Case 2	Not mentioned	Nivolumab, 1 dose	Carboplatin and Paclitaxel	Not mentioned	NA	NA	NA	None	NA	NA	Dead
	Case 3	Not mentioned	Nivolumab, 10 doses	Lobectomy, Cisplatin, Docetaxel, Carboplatin, Paclitaxel	28	SD	28	NA	Pneumonitis (III)	552	NA	Alive
	Case 4	Not mentioned	Nivolumab, 10 doses	Carboplatin, Alimta	24 ongoing	PR	NA	NA	Colitis (II) Rash (I)	460	NA	Alive
	Case 5	Not mentioned	Nivolumab, 17 doses	Carboplatin, Alimta, Docetaxel	34	PR	NA	NA	None	402	<400	Alive
	Case 6	Not mentioned	Pembrolizumab, 6 doses	Carboplatin, Paclitaxel	16	SD	NA	NA	None	517	NA	Dead
	Case 7	Not mentioned	Pembrolizumab, 7 doses	Carboplatin, Alimta, Trastuzumab, Pertuzumab	22	SD	NA	NA	Fatigue (I) Hypothyroidism	597	<20	Alive
	Case 8	Not mentioned	Nivolumab, 8 doses	Carboplatin, Alimta	16 ongoing	PR	NA	NA	Elevated TSH	NA	NA	Alive
	Case 9	Not mentioned	Nivolumab, 5 doses	Cisplatin, Etoposide	Not mentioned	PD	NA	NA	None	NA	NA	Dead
**Bari et al. 2019** [38]	Case 10	Not mentioned	Pembrolizumab, 2 doses	Erlotinib	Not mentioned	PD	NA	NA	None	NA	NA	Dead

	Case 11	Not mentioned	Nivolumab, 1 dose	Chemoradiation, Carboplatin, Paclitaxel	Not mentioned	PD	NA	NA	None	NA	NA	Dead
	Case 12	Not mentioned	Nivolumab, 13 doses	Mitomycin, Xeloda	26	PD	NA	NA	Hypothyroidism	262	<20	Dead
	Case 13	Not mentioned	Nivolumab, 12 doses	Regorafenib	25	SD	NA	NA	Fatigue (I)	431	NA	Dead
	Case 14	Not mentioned	Nivolumab, 12 doses	Radiofrequency ablation, TACE, Sorafenib	25 ongoing	PR	NA	NA	Hyperglycemia Hypothyroidism	120	<20	Alive
	Case 15	Not mentioned	Nivolumab, 11 doses	Sorafenib, Sunitinib, Everolimus, Axitinib	22	SD	NA	NA	Elevated TSH	376	<400	Dead
	Case 16	Not mentioned	Nivolumab, 10 doses	RCHOP, RICE, PBSCT, Revlimid, Rituximab	22	PD	NA	NA	None	285	<20	Dead
	Case 17	Not mentioned	Nivolumab, 10 doses	Vismodegib	24 ongoing	PR	NA	NA	Elevated TSH	370	<400	Alive
**Blanch-Lombarte et al. 2019 [39]**	Case 1	Axillary lymphadenectomy (before pembrolizumab)	Pembrolizumab (2 mg/kg IV, every 3 weeks; 12 cycles)	None	Not specified	CR	~9	None	None	544	Transient reductions in HIV-1 DNA (24–32%) at weeks 3 and 27, but no sustained decline	Alive

**Al Homsi et al. 2018 [40]**	Case 1	Cisplatin/etoposide chemotherapy and radiotherapy	Avelumab, 10 mg/kg, intravenous, (10 cycles)	None	Not mentioned	Positive response	NA	NA	Pneumonitis (IV) Leukopenia (II) Hemiparesthesia (II) Granulocytopenia (I) Anemia (I) Thrombocytopenia (I) Hyperglycemia (I) Elevated transaminase (I) Convulsions Dizziness	NA	<2,000	Alive
**Chang et al. 2018 [41]**	Case 1	Not mentioned	Nivolumab 3 mg/kg q2w, 7 doses	None	8	SD	8	NA	Pneumonitis (IV), Rash (II)	NA	Decreased by 1 log	Not reported
	Case 2	Not mentioned	Nivolumab, 3 doses	None	3	PD	3	NA	Pneumonitis (III)	NA	stable	Not reported

	Case 3	Not mentioned	Nivolumab, 4 doses	None	2	PD	2	NA	None	180	stable	Not reported
	Case 4	Not mentioned	Nivolumab, 14 doses	None	Not mentioned	CR	Not reached (>32 months)	NA	None	300	Decreased by 1 log	Not reported
	Case 5	Not mentioned	Nivolumab, 18 doses	None	Not mentioned	CR	>10	NA	Autoimmune diabetes (II)	391	stable	Not reported
	Case 6	Not mentioned	Nivolumab, 2 doses	None	Not mentioned	NA	1	NA	Hypothyroidism (II)	915	NA	Not reported
	Case 7	Not mentioned	Nivolumab, 3 doses	None	Not mentioned	PD	2.5	NA	None	63	NA	Not reported
	Case 8	Not mentioned	Nivolumab, 5 doses	None	Not mentioned	PD	2	NA	None	400	NA	Not reported
	Case 9	Not mentioned	Nivolumab, 6 doses	None	Not mentioned	PD	2	NA	None	374	stable	Not reported
	Case 10	Not mentioned	Nivolumab, 6 doses	None	Not mentioned	PD	3.5	NA	None	189	Decreased by 1 log	Not reported
	Case 11	Not mentioned	Nivolumab, 6 doses	None	Not mentioned	PD	3	NA	None	380	stable	Not reported
	Case 12	Not mentioned	Nivolumab, 9 doses	None	Not mentioned	SD	>10	NA	Pneumonitis (III)	194	stable	Not reported

	Case 13	Not mentioned	Nivolumab, 32 doses	None	Not mentioned	PD	>15	NA	None	189	Decreased by 0.5 log	Not reported
	Case 14	Not mentioned	Nivolumab, 1 dose	None	Not mentioned	NA	NA	NA	None	681	NA	Not reported
	Case 15	Not mentioned	Nivolumab, 3 doses	None	Not mentioned	PD	2	NA	Pneumonitis (III)	613	stable	Not reported
	Case 16	Not mentioned	Nivolumab, 2 doses	None	Not mentioned	PD	1	NA	None	280	NA	Not reported
**Galanina et al. 2018 [42]**	Case 1	Bortezomib, Lenalidomide	Nivolumab 3 mg/kg IV q2w	None	6.5+ , ongoing	SD	6.5+	Ongoing	No drug-related grade >2 toxicities	NA	29	Alive
	Case 2	Lenalidomide	Pembrolizumab 200 mg IV q3w	None	5+ , ongoing	PR	5+	Ongoing	No drug-related grade >2 toxicities	NA	92	Alive
	Case 3	Liposomal Doxorubicin, Lenalidomide, Paclitaxel, Bortezomib	Nivolumab 3 mg/kg IV q2w	None	3.5+ , ongoing	PR	3.5+	Ongoing	No drug-related grade >2 toxicities	NA	ND	Alive
	Case 4	None	Nivolumab 3 mg/kg IV q2w	None	6.5+ , ongoing	SD	6.5+	Ongoing	No drug-related grade >2 toxicities	NA	<20	Alive
	Case 5	None	Nivolumab 3 mg/kg IV q2w	None	6.5+ , ongoing	PR	6.5+	Ongoing	No drug-related	NA	118	Alive

									grade >2 toxicities			
	Case 6	None	Nivolumab 3 mg/kg IV q2w	None	5.5+ , ongoing	CR	5.5+	Ongoing	No drug-related grade >2 toxicities	NA	64	Alive
	Case 7	Liposomal Doxorubicin, Paclitaxel, Bortezomib	Nivolumab 3 mg/kg IV q2w	None	3.5+ , ongoing	SD	3.5+	Ongoing	No drug-related grade >2 toxicities	NA	84	Alive
	Case 8	Liposomal Doxorubicin	Nivolumab 3 mg/kg IV q2w	None	1.5+ , ongoing	PR	1.5+	Ongoing	No drug-related grade >2 toxicities	NA	1,210,000	Alive
	Case 9	Liposomal Doxorubicin	Nivolumab 3 mg/kg IV q2w	None	1.5+ , ongoing	PR	1.5+	Ongoing	No drug-related grade >2 toxicities	NA	ND	Alive
**Ostios-Garcia et al. 2018 [43]**	Case 1	Abacavir, dolutegravir, lamivudine	1st line, Pembrolizumab	None	3	SD	NA	NA	None	307	Undetectable (<20 copies/mL)	Not reported
	Case 2	Abacavir/lamivudine, dolutegravir, tenofovir disoproxil fumarate	2nd line, Nivolumab	None	2, discontinued due to progression	PD	NA	NA	Arthralgia (I) Headache Chest pain	NA	Undetectable	Not reported
	Case 3	Emtricitabine/tenofovir disoproxil	1st line, Pembrolizumab	None	3, ongoing	PR	NA	NA	Arthralgia (II)	140	42 copies/mL	Not reported

		fumarate, ritonavir, atazanavir										
	Case 4	Raltegravir, etravirine, darunavir	2nd line, Pembrolizumab	None	10, discontinued due to unrelated death	PR	NA	NA	Fatigue (I)	1229	Undetectable	Not reported
	Case 5	Emtricitabine/tenofovir disoproxil fumarate, dolutegravir	3rd line, Nivolumab	None	5, discontinued due to progression	SD	NA	NA	None	435	Undetectable	Not reported
	Case 6	Elvitegravir/cobicistat/emtricitabine/tenofovir alafenamide fumarate	1st line, Pembrolizumab	None	8 months, ongoing	PR	NA	NA	Arthralgia (II)	233	Undetectable	Not reported
	Case 7	Emtricitabine/tenofovir alafenamide fumarate, dolutegravir	2nd line, Pembrolizumab	None	3, discontinued due to progression	PD	NA	NA	None	305	<30 copies/mL	Not reported
	Case 1	None	Nivolumab 3 mg/kg Q2W	None	Not specified	PD	3	4	None	NA	Unknown	Dead
	Case 2	None	Ipilimumab 3 mg/kg Q2W	None	Not specified	PD	1	5	Hepatitis (I)	NA	Unknown	Dead
	Case 3	Carboplatin + Paclitaxel	Ipilimumab 3 mg/kg + Nivolumab 1 mg/kg Q2W	Carboplatin + Paclitaxel	Not specified	PD	2	6	None	NA	408 (6 months)	Dead
**Heppt et al. 2017 [44]**	Case 4	None	Ipilimumab 3 mg/kg + Nivoluma	None	Not specified	PD	5	9	Colitis (III)	NA	Undetectable	Dead

			b 1 mg/kg Q2W									
	Case 5	None	Ipilimumab 3 mg/kg + Nivolumab 1 mg/kg Q2W	None	Not specified	CR	9	9	Myositis (IV)	NA	Undetectable	Alive
	Case 6	None	Pembrolizumab 2 mg/kg Q2W	None	Not specified	PD	3	5	Fatigue (I)	NA	68 (6 months)	Dead
	Case 7	Dabrafenib + Trametinib	Pembrolizumab 2 mg/kg Q2W	Dabrafenib + Trametinib	Not specified	NA	NA	NA	None	NA	Undetectable	Alive
	Case 8	Vemurafenib	Ipilimumab 3 mg/kg Q2W	Vemurafenib	Not specified	PD	1	2	None	NA	Unknown	Dead
	Case 9	Dacarbazine, Vemurafenib	Ipilimumab 3 mg/kg Q2W	Dacarbazine, Vemurafenib	Not specified	PR	5	4	None	NA	Undetectable	Dead
	Case 10	Liposomal Doxorubicin	Pembrolizumab 2 mg/kg Q2W	Liposomal Doxorubicin	Not specified	NA	NA	NA	Pneumonitis (I)	NA	54 (3 months) 102 (6 months) 40 (9 months) <21 (12 months) |	Alive
**Davar et al. 2015 [45]**	case 2	Interferon, Ipilimumab, Dabrafenib/Trametinib	Pembrolizumab, 2 mg/kg IV q3w, 2 doses	None	<2	PD	NA	Decreased	NA	NA	NA	Dead
**Burke et al. 2011 [46]**	Case 1	IL-2 therapy	Ipilimumab	None	Not specified	PD	NA	Still alive at follow-up	Self-limited (II)	15	Undetectable	Alive at the time

												of re-port-ing

Disclaimer/Publisher's Note: The statements, opinions, and data contained in all publications are solely those of the individual author(s) and contributor(s) and not of MDPI and/or the editor(s). MDPI and/or the editor(s) disclaim responsibility for any injury to people or property resulting from any ideas, methods, instructions, or products referred to in the content.
